# Molecular Determinants of the Cellular Entry of Asymmetric Peptide Dendrimers and Role of Caveolae

**DOI:** 10.1371/journal.pone.0147491

**Published:** 2016-01-20

**Authors:** Prarthana V. Rewatkar, Harendra S. Parekh, Marie-Odile Parat

**Affiliations:** The University of Queensland, School of Pharmacy, 20 Cornwall Street, Woolloongabba, QLD 4102, Australia; University of British Columbia, CANADA

## Abstract

Caveolae are flask-shaped plasma membrane subdomains abundant in most cell types that participate in endocytosis. Caveola formation and functions require membrane proteins of the caveolin family, and cytoplasmic proteins of the cavin family. Cationic peptide dendrimers are non-vesicular chemical carriers that can transport pharmacological agents or genetic material across the plasma membrane. We prepared a panel of cationic dendrimers and investigated whether they require caveolae to enter into cells. Cell-based studies were performed using wild type or caveola-deficient i.e. caveolin-1 or PTRF gene-disrupted cells. There was a statistically significant difference in entry of cationic dendrimers between wild type and caveola-deficient cells. We further unveiled differences between dendrimers with varying charge density and head groups. Our results show, using a molecular approach, that (i) expression of caveola-forming proteins promotes cellular entry of cationic dendrimers and (ii) dendrimer structure can be modified to promote endocytosis in caveola-forming cells.

## Introduction

**Fig 1:**
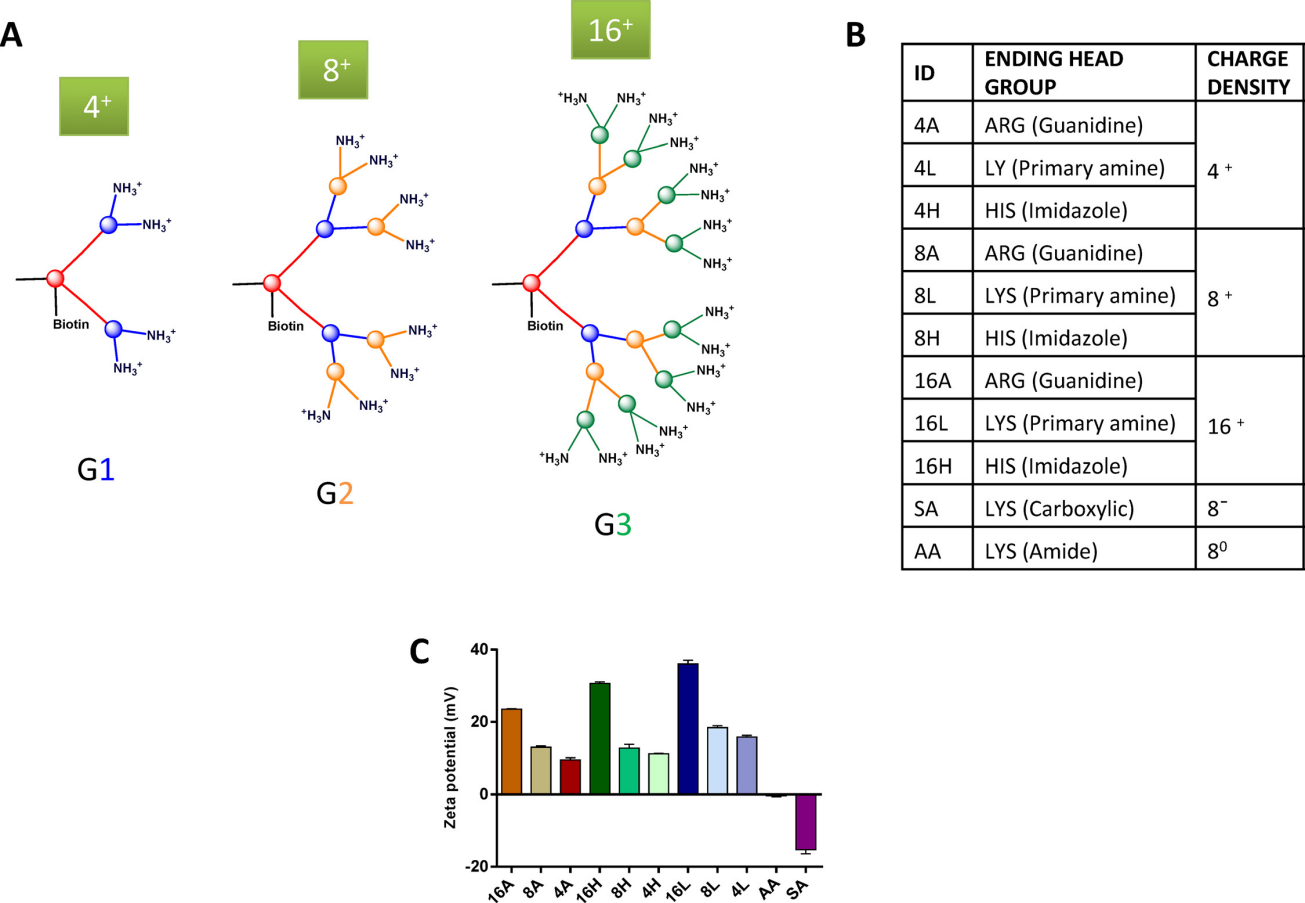
Dendrimers used in this study. A) General structure of 4 (G1 generation), 8 (G2 generation) and 16 (G3 generation) charged cationic dendrimers. B) ID of dendrimers and their ending head groups and charge. C) Average zeta potential of the biotinylated dendrimers at 1mg/mL in 10 mM NaCl solution. The mean ± S.E.M. (n = 3 separate measurements) is shown; A = Arginine, L = Lysine, H = Histidine, AA = Neutral (Amide) and SA = Anionic (carboxylic) head group.

Dendrimers have great potential as multifunctional nano-scale devices [1–3]. They are built-up layer upon layer, forming so-called ‘generations’, terminating with surface groups which are typically basic in nature e.g. amine [4], imidazoles [5], guanidines [6]. The unique architecture and surface of dendrimers determines their capacity to interact with typically non-internalizable molecules (e.g. genes) as well as their ability to be further polymerized or functionalized [7]. Asymmetric peptide based cationic dendrimers represent a family of highly branched chemically-derived vectors, comprising a functionalised core from which branches extend. The precise cationic surface head group chemistry enables the strong electrostatic interaction with cell membranes and substantially increases the binding strength to biological targets. Asymmetric dendrimers (G1, G2 and G3, Fig 1A) are biocompatible and less toxic compared to high and low generation commercial PAMAM dendrimers [8,9].

Caveolae are plasma membrane subdomains enriched in cholesterol that appear as flask shaped, little caves, that are not clathrin coated, with a neck size of 30–60 nm depending on the cell type [10]. Membrane-inserted proteins of the caveolin family and cytoplasmic proteins of the cavin family are required for caveola formation, and genetic ablation of either caveolin-1 or cavin-1 results in a loss of caveolae [11–13]. One of the multiple roles of these organelles is to participate directly in endocytosis [14]. Furthermore, caveolin-1 expression has been show to prevent fluid phase endocytosis via the cdc42-clic pathway [15]. We have recently reviewed the literature claiming caveolar entry for a variety of non-viral vectors [16]. A majority of studies employ a pharmacological approach, relying on disruption of cholesterol-rich domains by cholesterol-binding agents to decrease intracellular delivery, however this is likely to disrupt the cell membrane and endocytic processes in multiple ways that lack caveola specificity. In the current study we investigated whether the internalization of a panel of cationic peptide based dendrimers required the presence of caveolae using mouse embryo fibroblasts isolated from gene-disrupted mice lacking one of the two proteins essential for caveola formation, namely caveolin-1 or cavin-1 null cells compared to cells isolated from their wild type counterparts.

## Materials and Methods

### Reagents

All fluorenylmethyloxycarbonyl (Fmoc) amino acids and Rink amide resin (200–400 mesh) were purchased from NovaBiochem (NSW, Australia). Peptide grade *N*, *N*-dimethylformamide (DMF) was from Merck (NSW, Australia). Trifluoroacetic acid (TFA), *O*-benzotriazole-*N*,*N*,*N'*,*N'*-tetramethyl-uronium-hexafluoro-phosphate (HBTU), *N*, *N-*diisopropylethylamine (DIPEA), dichloromethane (DCM), triisopropylsilane (TIPS) and piperidine, 3-(4,5-dimethylthiazoll-2-yl)-2,5-diphenyltetrazolium bromide (MTT), dimethyl sulfoxide (DMSO) and gelatin were obtained from Sigma-Aldrich (Castle Hill, NSW, Australia). *D*-biotin was purchased from Shen Zhen Inno Syn Biotech Co., Ltd (Shenzhen, China). Cy3-avidin was from Invitrogen life technologies (Victoria, Australia). Dulbecco’s modified Eagle’s medium (DMEM), penicillin/streptomycin solution, trypsin, and foetal bovine serum (FBS) were purchased from Invitrogen (Life Technologies, Mulgrave, VIC, Australia). Mounting medium with 4',6-diamidino-2-phenylindole (DAPI) was purchased from Vector Laboratories (Burlington, Canada). Rabbit anti-caveolin-1 polyclonal antibody was from BD Biosciences (NSW, Australia) and Alexa fluor® 488 goat anti-rabbit antibody from Thermofisher Scientific (VIC, Autralia).

### Cell culture

Immortalized Mouse Embryo Fibroblasts (iMEF) isolated from wild type (WT), caveolin-1 gene-disrupted (Cav-1 KO) and PTRF gene-disrupted (PTRF KO) mice, a generous gift from Prof. Robert G. Parton, were previously generated [12,17,18]. They were grown in DMEM medium supplemented with 5% (v/v) FBS, 100 IU/mL penicillin, 100μg/mL streptomycin at 37°C in a humidified atmosphere with 5% CO_2_.

### Synthesis of asymmetric amino acid based peptide dendrimer

In this study, dendrimers were designed and synthesized using solid phase peptide synthesis (SPPS). Dendrimers were constructed with various charge (cationic, anionic or neutral), charge density (4^+^, 8^+^, 8^-^, 8 neutral or 16^+^ charged) and head groups (arginine, lysine or histidine). Anionic and neutral dendrimers were capped with succinic anhydride and acetic anhydride, respectively. All the dendrimers were prepared on insoluble solid support (rink amide resin, loading capacity; 0.47 or 0.79) with sequential addition of amino acid building blocks and side arm biotinylation. The percentage of amino acid coupling was monitored by UV spectrophotometry (Varian Cary 50 UV-Vis).

The resulting crude peptides were purified by preparative RP-HPLC, Waters (USA), system (Model 600 controller, 2996 photodiode array detector, Elite Alltech degassing system and MassLynx^TM^ software) with a C_18_ column (GraceVydac; particle size 10μm pore size, id = 22mm x 250mm). To assess analytical purity of dendrimers, reverse phase high performance liquid chromatography (RP-HPLC), Shimadzu (Japan) system controller- CBM-20A, pump A- LC-10AD, autosampler–SIL-10AXL, a variable wavelength UV/vis detector, degasser–DGU As Prominence) with a C_18_ column (GraceVydac; particle size 5μm pore size, id = 4.6mm x 250mm, pore size: 300Å) was employed. The detection wavelength in both cases was 219 nm. The mobile phase employed was: Solvent A; (100% H_2_O), solvent B; (90% CH_3_CN_(aq)_), with a flow rate of 10 mL/min used for preparative RP-HPLC. In the case of analytical RP-HPLC a flow rate of 1 mL/min was used with an injection volume of 10μL and the following gradient was employed (from 0–100% B with over 20 min): solvent A- distilled water with 0.1% v/v TFA and solvent B- CH_3_CN_(aq)_ with 0.1% v/v TFA. A blank chromatogram reading zero was run between each sample. Liquid chromatography-mass spectrometry was performed on an Applied Biosystem/MDS Sciex Q-TOF LC/MS/MS system (Agilent technologies, Australia) to visualize the molecular ion peak in order to verify identity and purity of the dendrimers.

### Measurement of Zeta potential

The particle charge measurements were conducted using a dynamic light scattering phase analysis with Malvern ZetaSizer Nano ZS. The sample were prepared in 10 mM NaCl, transferred to a zeta cell (Malvern Instruments, DTS1060) and measured at 25°C with an applied voltage of 150 V.

### *In vitro* microscopic fluorescence internalization study

WT, Cav-1 KO and PTRF KO iMEF cells were seeded on 0.1% gelatin-coated glass coverslips at a density of 25,000 cells per well in 24 well plates. After 24 h cells were incubated with different concentrations of biotinylated dendrimers (0, 10, 20, 50 and 100 μg/mL) for 12 h. The cells were then rinsed with phosphate buffered saline solution (PBS) and fixed with ice cold methanol for 20 min at room temperature. The cells were washed thrice with PBS and permeabilized for 10 min with PBS containing 0.1% (v/v) Triton X100. After three washes with PBS, cells were incubated with Cy3-streptavidin (3:1000 in PBS) for 10 min. Cells were washed and mounted on microscopic slides with DAPI-containing mounting medium to stain nuclei for fluorescence imaging analysis. The number of Cy3-fluorescent cells and the number of nuclei per field were counted manually in five different fields per slide using Image J, and results were expressed as the percentage of dendrimer-positive cells. The average number of cells per captured image was ≥ 50. In some experiments (with 16 charged dendrimers at all concentrations), where 100% cells were Cy3-positive in WT and KOs, the fluorescence intensity per cell was quantified using Image J and expressed as arbitrary units (AU) per cell. Each experiment was separately repeated three times.

### Acid wash

In some experiments, acid wash was performed as follows to remove extracellularly membrane bound dendrimers before fixation. Biotinylated dendrimer treated WT, Cav-1 KO and PTRF KO iMEF cells after 12 h of incubation, were washed twice for 30 seconds with acid/glycine buffer of pH range 2.2–2.5 (100 mL of 0.5M glycine + 22 mL 0f 0.5M HCl diluted upto 400mL water) prior to fixation with ice cold methanol for 20 min at room temperature. The cells were washed thrice with PBS and permeabilized for 10 min with PBS containing 0.1% (v/v) Triton X100. After three washes with PBS, internalized dendrimers were labelled/stained with Cy3-streptavidin (3:1000 in PBS) for 10 min. Cells were washed and mounted on microscopic slides with DAPI-containing mounting medium to stain nuclei. The fluorescence imaging analysis were performed same as before in *in-vitro* microscopic fluorescence internalization study. We verified the efficiency of this method to remove extracellularly bound dendrimers in an experiment where internalization was inhibited using 10 or 100 mM of sodium azide for 30 minutes, cells exposed to 50 μg/ml 16^+^ arginine dendrimers for 30 additional minutes, then acid wash performed before fixation and staining as described above: when internalization was prevented, the acid wash eliminated the entirety of the red staining present at the surface of the cells. In contrast, acid wash did not alter red fluorescent staining when internalization was allowed to proceed in absence of Na azide (data not shown).

### MTT assay

The effect of biotinylated 16 charged dendrimers on WT and KO cells was assessed using a 3-(4,5-dimethyl-2-thiazoyl)-2,5-diphenyl-2H-tetrazolium bromide (MTT) assay. Briefly, WT and KO cells were seeded at 7,000 and 13,000 cells per well, respectively. The cells were seeded in 100μL of DMEM (5% serum) in a 96 well plate. After overnight incubation, cells were treated with 100 μg/mL 16 charged dendrimers for 12 h. Each sample was tested in triplicate. After 12 h, the medium was replaced with 100 μL of MTT solution (0.5mg/mL) and incubated for an additional 3 h. The medium was aspirated, and 100μL dimethyl sulfoxide (DMSO) were added to dissolve formazan crystals. After 5 min of incubation, the absorbance at 590 nm was measured. The results were expressed as percent of the viability of control cells ± SEM.

### Statistical analysis

All the results were reported as mean ± SEM using GraphPad Prism (V6.0 for windows). *p* values < 0.05 were considered significant.

## Results

### Synthesis and characterization of dendrimers

Asymmetric cationic, anionic and neutral capped peptide based dendrimers (Fig 1A and 1B) were synthesized using Fmoc SPPS. After purification of the biotinylated reaction product (using preparative RP-HPLC), all dendrimers were obtained in high yield (>65%) and the final product was a fluffy white fibrous solid. Single peak purity was confirmed using analytical RP-HPLC (S1 Fig) whereas the molecular weight of each dendrimer was confirmed by HR-MS; *m/z* (range: 900–27,000 [M+H]^+^). The zeta potential of each dendrimer was measured in 10 mM NaCl with a Malvern ZetaSizer Nano ZS. The average zeta potential (mV) values (Fig 1C) were around 22–37 for 16^+^, 12–20 for 8^+^, 9–15 for 4^+^, -0.3313 for neutral dendrimer and -15.233 for anionic dendrimer at pH 6.6–6.8 range. An increase in the measured value of zeta potential was observed and consistent with the increased number of positively charged (N^+^ groups) surface groups.

### Dendrimer charge influences entry in wild type cells

**Fig 2:**
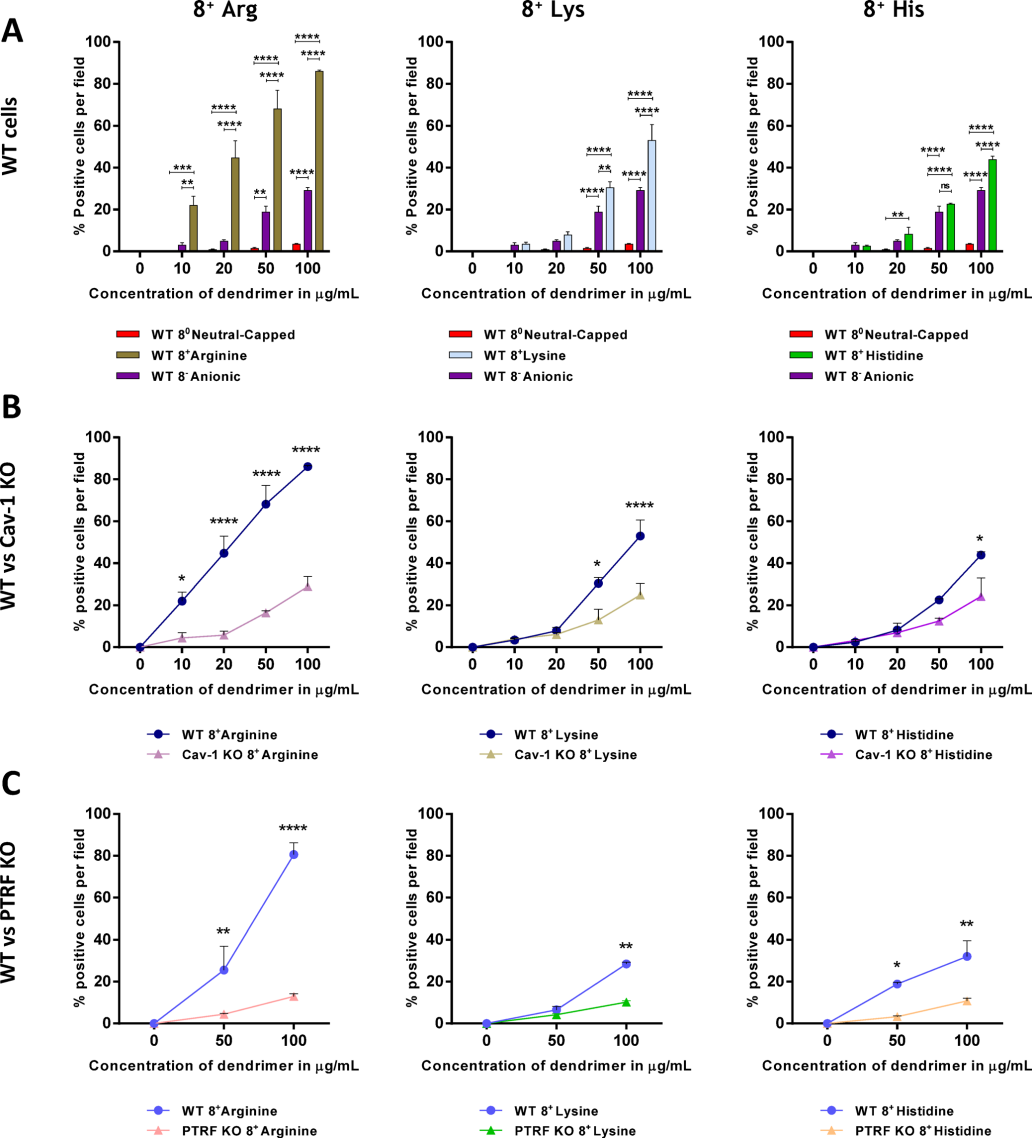
Effect of charge and role of caveolae in dendrimer internalization. A) Cells were exposed for 12 h to 8 charged cationic, anionic and neutral capped dendrimers and internalization was assessed after fixation using fluorescence microscopy. Results are reported as % biotin-positive cells in five fields per slide as determined using Image J and shown as mean ± S.E.M. (n = 3 separate experiments), **p<0.01; ***p<0.001; ****p<0.0001. B) WT and caveolin-1 KO iMEFs were exposed to 8 charged cationic dendrimer with either Arg, his or Lys head groups. Internalization was assessed using fluorescence microscopy, is reported as % biotin-positive cells and shown as mean ± S.E.M. (n = 3 separate experiments), *p<0.05; ****p<0.0001. C) WT and PTRF KO iMEFs were exposed to 8 charged cationic dendrimer with either Arg, his or Lys head groups. Internalization was assessed using fluorescence microscopy, is reported as % biotin-positive cells and shown as mean ± S.E.M. (n = 3 separate experiments), *p<0.05; ***p<0.001; ****p<0.0001.

We first analysed the internalization of 8 charged or neutral dendrimers in WT iMEFs. Dendrimers were either cationic-amine terminated, anionic-carboxylic acid terminated or neutral acetylated amines. Internalization was allowed for 12 h and detected after fixation using Cy3-streptavidin via fluorescence imaging (Fig 2A). Cationic dendrimers were internalized efficiently and their cellular entry was dose-dependent. Cationic dendrimers internalized significantly more compared to anionic and neutral dendrimers in WT fibroblasts. This was apparent with all three head groups tested (Arg, Lys and His). Moreover, anionic dendrimers were internalized to a significantly greater extent than neutral dendrimers, which were barely detected within the cells at the concentrations tested.

### The presence of caveolae promotes cellular entry of cationic dendrimers

We next compared the cellular uptake of cationic dendrimers in caveola-forming cells and cells devoid of caveolae (Fig 2B and 2C). With all head groups (Arg, Lys and His), cellular entry was higher in WT iMEFs than in caveolin-1-KO iMEFs (Fig 2B) or PTRF-KO iMEFs (Fig 2C) and the difference was statistically significant. Of note, cellular entry of cationic dendrimers was not totally abolished in KO cells, indicating that in addition to a pathway requiring caveolae, cells utilise another mechanism or route to take up the dendrimers we tested.

### The difference between positively and negatively charged dendrimer entry is abolished in cells devoid of caveolae

**Fig 3:**
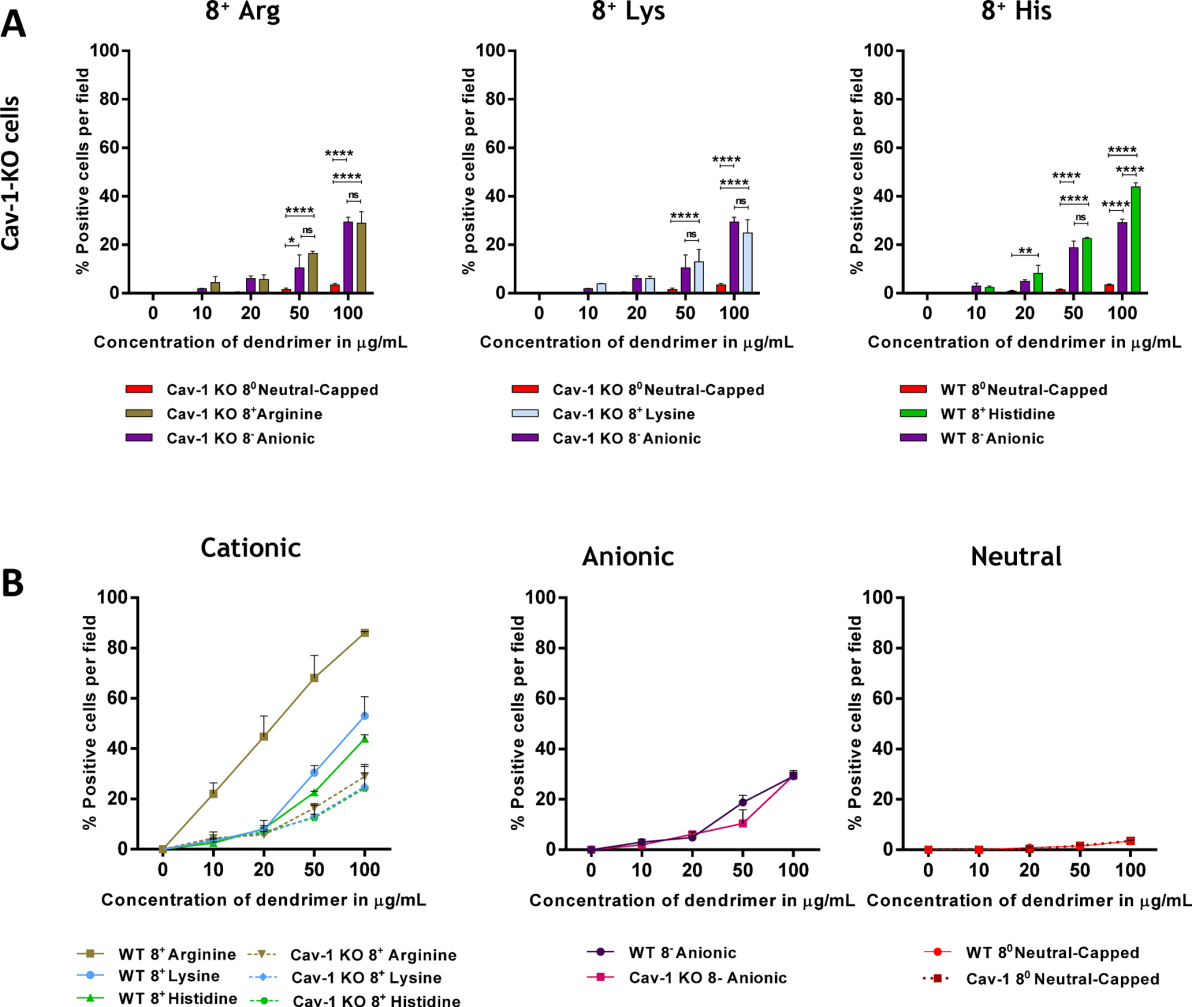
Positive charge-specific entry is abolished in cells devoid of caveolae. A) Caveolin-1 KO cells were exposed for 12 h to 8 charged cationic, anionic and neutral capped dendrimers and internalization was assessed after fixation using fluorescence microscopy. Results are reported as % biotin-positive cells in five fields per slide as determined using Image J and shown as mean ± S.E.M. (n = 3 separate experiments), ns; not significant *p<0.05; **p<0.01; ****p<0.0001. B) Cationic, anionic and neutral dendrimers internalization after 12 h was assessed in WT and caveolin-1 KO cells using fluorescence microscopy. Results are reported as % positive red cells and shown as mean ± S.E.M. (n = 3 separate experiments).

We measured the entry of cationic, anionic and neutral dendrimers in caveolin-1 KO iMEFs (Fig 3A). There was no difference between anionic and cationic dendrimer entry in the absence of caveolae. This shows that the advantage given to positively charged dendrimers over negatively charged dendrimers relies on the presence of caveolae. In contrast, the difference in internalization between negatively charged and neutral dendrimers was unaltered by the lack of caveolae (Fig 3A). The finding that anionic dendrimers enter cells via a caveola-independent pathway was confirmed by the superimposable dose response in WT and caveolin-1-KO iMEFs (Fig 3B). Taken together, these results show that cationic dendrimers rely on the presence of caveolae to enter cells and that their entry is not null in cells devoid of caveolae. They further indicate that anionic dendrimers do not enter cells using a caveola-dependent pathway and that neutral dendrimers do not enter cells at all.

### Positive charge density promotes cellular internalization

**Fig 4:**
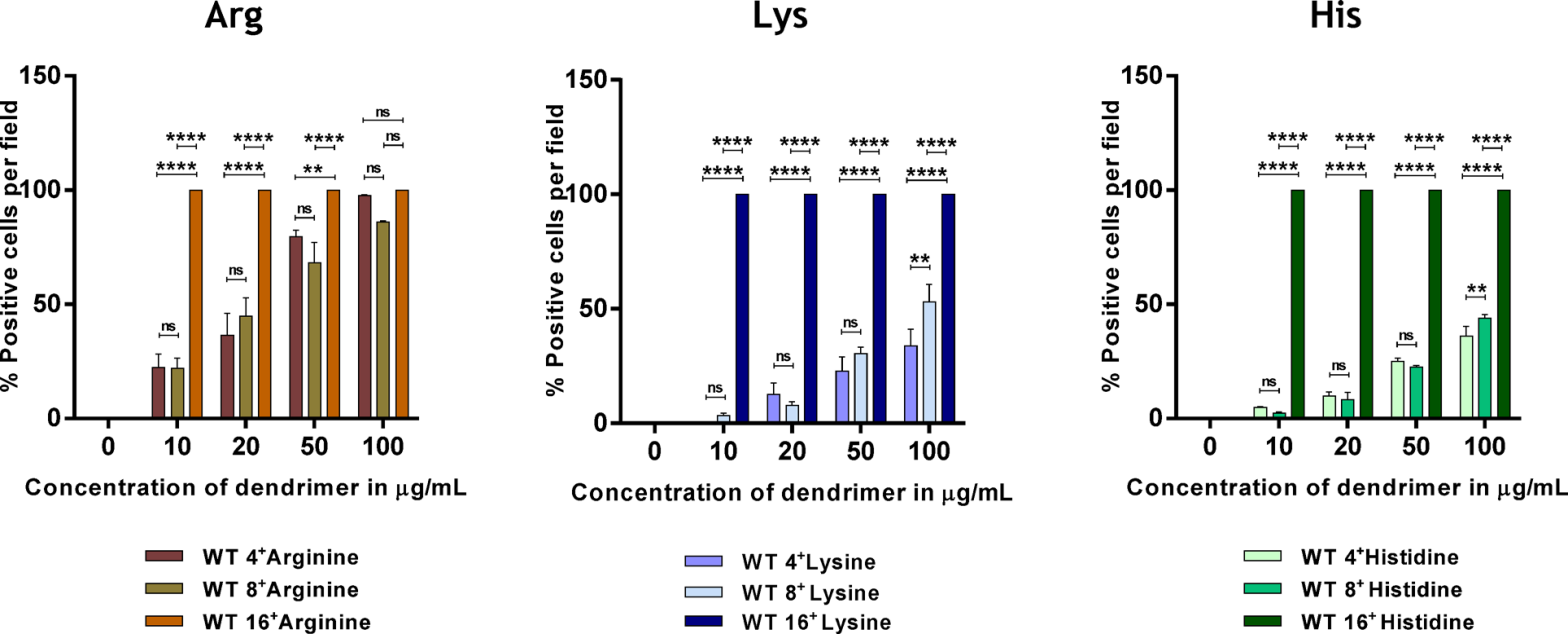
Effect of positive charge density on dendrimer cellular entry. WT cells were incubated with 4, 8 and 16 charged cationic dendrimers for 12 h. Internalization was assessed using fluorescence microscopy. Results are reported as % biotin-positive red and shown as mean ± S.E.M. (n = 3 separate experiments), ns; not significant, **p<0.01; ****p<0.0001.

Identical dendrimers of different generation (1 to 3) were compared to explore the role of charge density in *in vitro* cellular internalization using fluorescence imaging microscopy. An increase in uptake with increased charge density was observed in iMEF WT cells (Fig 4). The 16^+^ dendrimers were detected in 100% of cells at all tested dendrimer concentrations irrespective of head groups, and their internalization was significantly higher than that of 4^+^ and 8^+^ dendrimers. However, not much difference was observed between 4^+^ and 8^+^ dendrimers internalization (Fig 4).

### Effect of head groups chemistry

**Fig 5:**
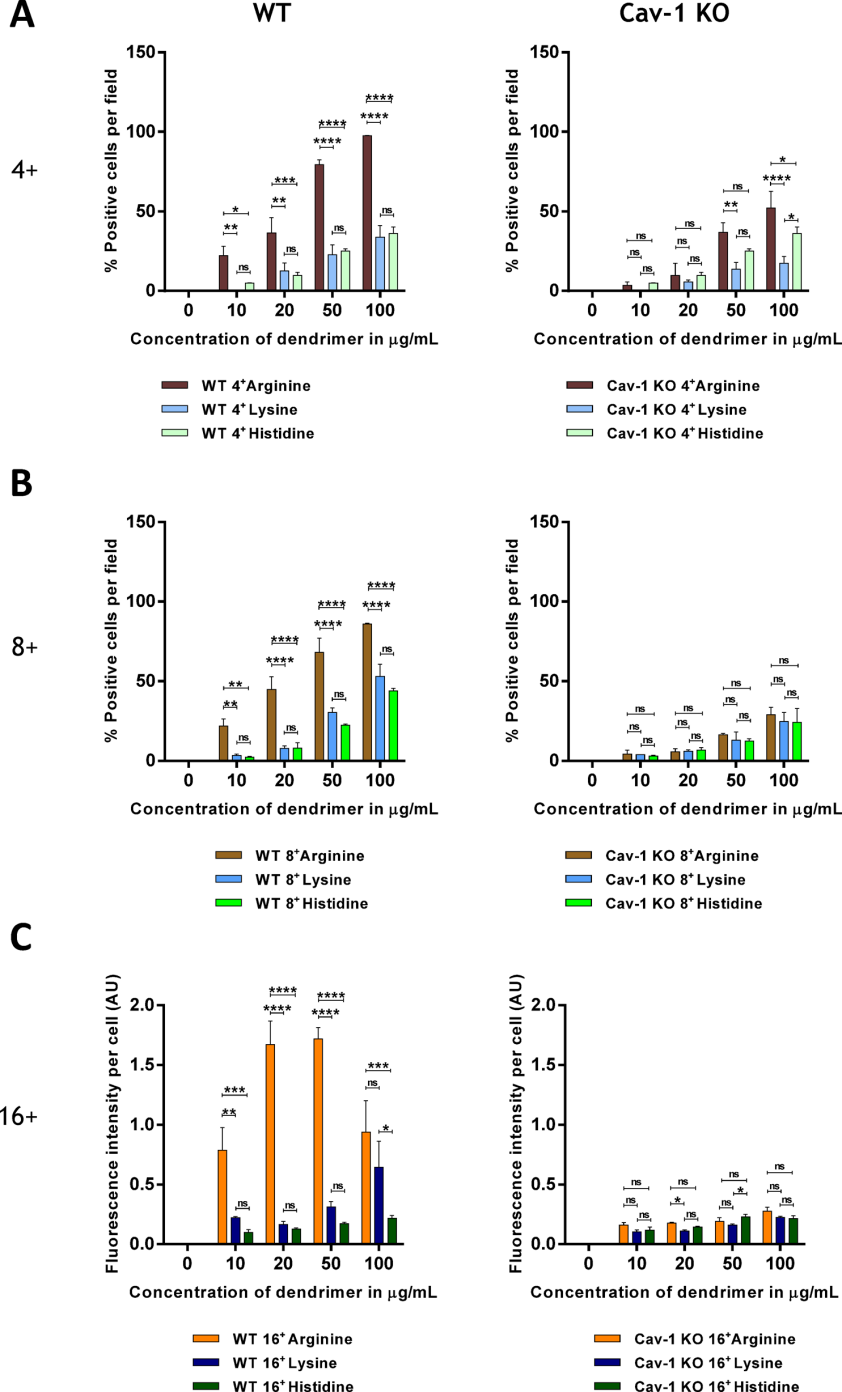
Arginine head groups promote entry of dendrimers in cells forming caveolae. WT and caveolin-1 KO MEFs were incubated for 12 h with (A) 4 charged (B) 8 charged and (C) 16 charged cationic dendrimers. Cells were imaged using fluorescence microscopy after fixation. A, B) Results are reported as % positive red cells and shown as mean ± S.E.M. (n = 3 separate experiments), ns; not significant, *p<0.05; **p<0.01; ***p<0.001; ****p<0.0001. C) Results are reported as fluorescence intensity per cell as measured using Image J, expressed as arbitrary units (AU) and shown as mean ± S.E.M. (n = 3 separate experiments), ns; not significant, *p<0.05; **p<0.01; ***p<0.001; ****p<0.0001.

The effect of head groups of dendrimers on *in vitro* cellular uptake was determined in a set of experiments involving WT and caveolin-1-KO cells. The 4^+^ and 8^+^ Arginine head group dendrimers internalized significantly more than their Histidine and Lysine counterparts (Fig 5A and 5B). There was no statistically significant difference between the internalization of Histidine and Lysine head group dendrimers. All 16^+^ dendrimers internalized in 100% of WT and Cav-1-KO cells (data not shown). Thus, to unveil a possible difference in internalization, per cell fluorescence intensity was measured using image J, and expressed as arbitrary units (Fig 5C). A significant difference in cellular uptake was observed in WT cells where Arginine dendrimers internalized more than Lysine or Histidine head group dendrimers. We further noted that internalization efficiency was only dose dependent until 50 μg/ml dendrimer concentration, with 100 μg/ml leading to lower fluorescence intensity per cell than 50 μg/ml in WT cells (Fig 5C). We verified using a MTT viability assay that this was not due to toxicity at the highest concentration of 16^+^ Arginine dendrimers (S2 Fig) and detected slight toxicity (> 94% survival) only with gene-disrupted cells. The advantage in uptake provided by Arginine head groups was largely reduced (4^+^) or abolished (8^+^ and 16^+^) in caveolin-1 KO cells (Fig 5). This strongly suggests that Arginine head groups promote entry of asymmetric dendrimers via a pathway that requires the presence of caveolae.

### Characterization of the endocytosis

**Fig 6:**
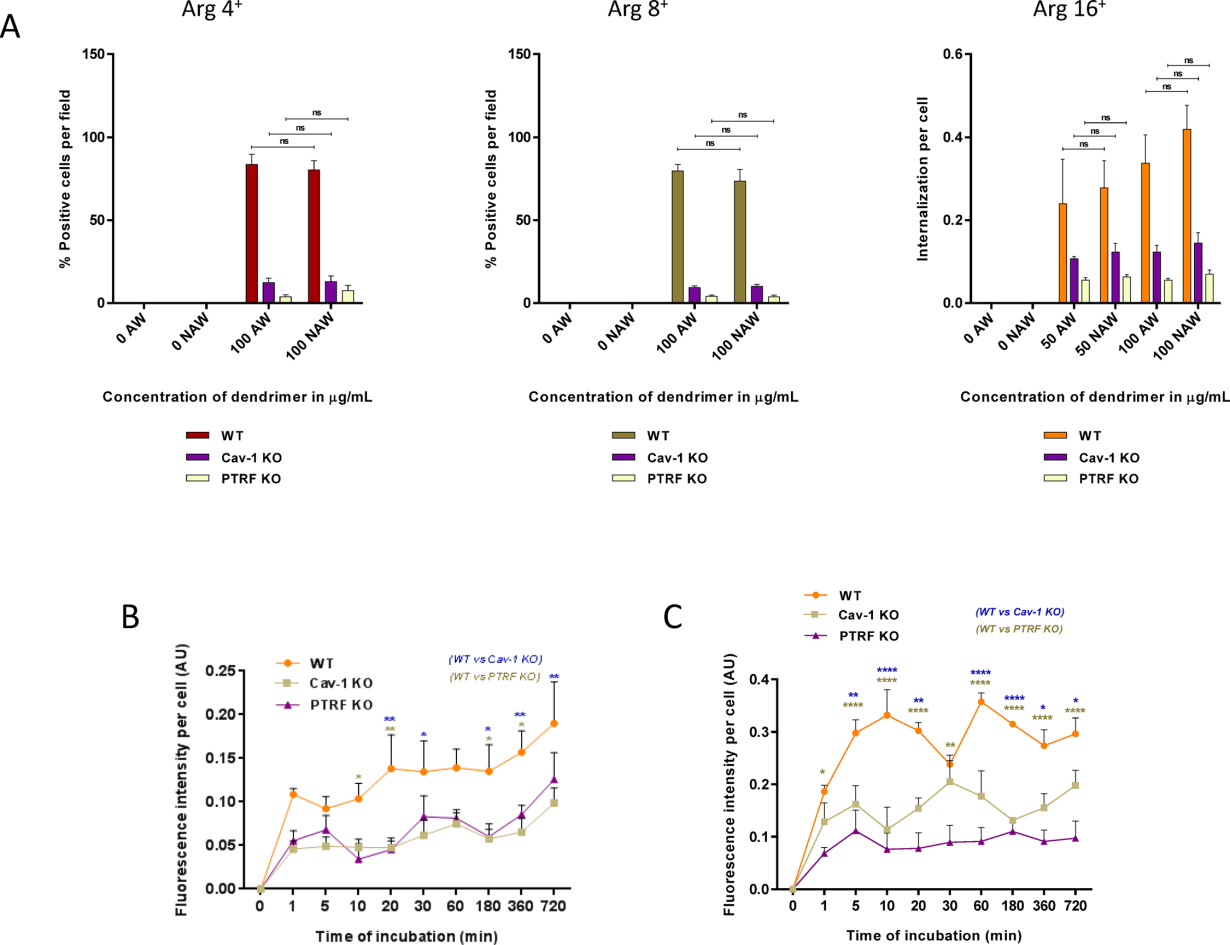
Characterization of dendrimer endocytosis. A) WT, caveolin-1 KO and PTRF- KO cells were incubated with 4, 8 and 16 charged cationic arginine dendrimer at concentrations 0, 50 and 100 μg/ml as indicated for 12 h. Cells were subjected to an acid wash (AW) to remove surface bound dendrimers, or washed with control buffer (No acid wash, NAW). Dendrimer endocytosis was assessed using fluorescence microscopy after fixation. For 4 and 8 charged dendrimers, results are reported as % biotin-positive red cells and shown as mean ± S.E.M. (n = 3 separate experiments), ns; not significant. For 16 charged dendrimers, results are reported as fluorescence intensity per cell expressed in arbitrary units (AU) and shown as mean ± S.E.M. (n = 3 separate experiments), ns; not significant. B) Kinetics of internalization of 16 charged cationic arginine dendrimer 50 μg/ml in WT, caveolin-1 KO and PTRF- KO cells was assessed using wild field fluorescence microscopy. Results are reported as fluorescence intensity per cell expressed in arbitrary units (AU) and shown as mean ± S.E.M. (n = 3 separate experiments), ns; not significant, *p<0.05; **p<0.01. C) Kinetics of internalization of 16 charged cationic arginine dendrimer 50 μg/ml in WT, caveolin-1 KO and PTRF- KO cells was assessed using confocal fluorescence microscopy. Results are reported as fluorescence intensity per cell expressed in arbitrary units (AU) and shown as mean ± S.E.M. (n = 3 separate experiments), ns; not significant, *p<0.05; **p<0.01; ****p<0.0001.

We verified using confocal microscopy that the red fluorescent granular staining observed in our experiments was intracellular (S3 Fig). Furthermore, to eliminate the possibility of measuring surface binding at the membrane rather than endocytosis, we performed acid wash experiments using Arginine head group dendrimers and WT, Cav-1-KO and PTRF-KO cells (Fig 6). There was no statistically significant difference between acid washed cells and non-acid washed cells for each group, confirming that the measured difference in fluorescence staining between caveola-forming and caveola-devoid cells is due to altered endocytosis. Lastly, we performed a time course experiment fixing the cells after 1 to 720 minutes of exposure to the 16 charged arginine dendrimer (Fig 6B and 6C). Increased uptake in the wild type cells was apparent as early as 1 min and the same trend was observed using confocal of wild field fluorescence microscopy.

## Discussion

In the current study, we examined the mechanism of internalization of a panel of asymmetric amino acid based peptide dendrimers using a unique approach relying on genetic ablation of caveolae, namely cultured cells isolated from caveolin-1 or PTRF knockout mice and compared to their wild type counterparts. A review of the literature [16] revealed that the vast majority of studies claiming entry of a delivery agent via caveolae employed chemical or pharmacological tools, and most frequently membrane cholesterol-sequestering agents such as cyclodextrin. Our study employs cells lacking caveolae via genetic ablation of one out of two distinct caveola-forming proteins. A disadvantage of long term ablation of a pathway is that it may allow cells to develop compensatory pathways, and short term siRNA down-regulation of either caveolin-1 or PTRF could be proposed to investigate whether caveolae play a role in dendrimer internalization. However short term down regulation of the expression of these proteins would only lead to a reduction, instead of total ablation, of caveola number, and may not be efficient especially in the case of caveolin-1 which is a long-lived protein [19]. Studying gene-disrupted cells thus seems the optimal approach four our study. In addition, the dendrimers that we study are small (sub-nm) sized [20,21] and therefore compatible with caveola entry via the 30–60 nm neck size of caveolae [22,23]. It is important to note that our experimental design demonstrates that the internalization pathway requires the existence of caveolae rather than directly proving entry via caveolae. It has been documented that caveolin-1 expression level regulates cdc42-mediated fluid phase endocytosis via the CLIC pathway due to its ability to function as a guanine nucleotide dissociation inhibitor and inhibit cdc-42 activation [15]. However in this study, silencing caveolin-1 increased rather than decreased endocytosis, which is opposite to the decreased dendrimer internalization observed in absence of caveolin-1 in our experiments. Other changes related to the absence of caveolae and caveola-forming proteins, such as changes in cholesterol distribution and plasma membrane composition, could indirectly affect dendrimer internalization.

Employing capped control dendrimers that are either anionic or neutral unveiled that anionic dendrimers do not enter cells via a mechanism requiring caveolae while neutral dendrimers do not enter cells at all. Positive charges have been shown to promote cellular entry in other systems [24,25], presumably because of an electrostatic interaction of positive particles with the negatively charged outer plasma membrane [26]. However, after reviewing a variety of positively charged particles such as quantum dots, polystyrene, chitosan, it was concluded that there is no particular route of endocytosis that can be ascribed preferentially to positively charged particles in non-phagocytic cells [26]. In our experiments, internalization of cationic dendrimers was very similar to that of anionic dendrimers in caveola-null cells, indicative of a route of entry which allows residual internalization in the absence of caveolae. Our results contrast with those from Perumal *et al*. who showed that of several FITC labelled PAMAM dendrimers carrying different charges (amine, carboxyl or hydroxyl surface functionality), only anionic dendrimers entered via caveolae. A major limitation of that study however, was the sole use of filipin to define caveola-mediated endocytosis [27]. Early literature studying the distribution of anionic sites on cell surface made use of *in vivo* or *ex vivo*, *in situ* administration of cationized ferritin probes followed by detailed analysis of electron micrographs. Results varied depending on the cell type examined [28]. In microvascular beds, cationic ferritin was shown to bind to plasmalemma proteoglycans and sialoglycoproteins, but to avoid for the most part the membrane of caveolae and their stomatal diaphragms [29,30]. A model was proposed whereby caveolae lacked anionic sites and thus favoured the uptake and transport of anionic molecules [28]. This in in apparent contrast to our results, and raises the possibility that the absence of caveolin-1, PTRF and/or caveolae may modulate cationic dendrimer endocytosis via alteration of the surface molecules present on the plamsalemma, and their distribution.

The fact that the difference between positively and negatively charged dendrimer internalization is abolished in KO cells would indicate that positive charges confer some entry specificity related to the presence of caveolae. This might be depending on the nature of the particle studied, because in their study of polyplexes, lipoplexes and lipopolyplexes, Billiet *et al*. showed that positively charged complexes entered cells via clathrin coated pits whereas negatively charged complexes entered cells via caveolae [25]. This was determined via co-localization studies in fluorescently tagged caveolin-1-expressing cells. The authors further showed that the transfection efficiency correlated with the endocytic pathway employed by the complexes rather than with the uptake efficacy [25], so that complexes entering via caveolae were weakly internalized but provided higher transfection efficiency. It would be interesting to test the internalization of the dendrimers studied here complexed to plasmid DNA, and their transfection efficiency. It is important to note that in the present study, the carriers were labelled with biotin but had no cargo, and negatively charged plasmid DNA would change the net charge of particles presenting to caveolae, thereby modulating their cellular entry capacity.

Our finding that arginine head groups promote cellular entry in cells forming caveolae is entirely novel. Arginine rich peptides, including natural linear peptide and synthetic branched chain peptides, are known to have the ability to translocate through cell membranes [31,32]. It has been suggested that the guanidine function of arginine play an important role in internalization possibly via hydrogen bonding with the phospholipids of the lid bilayers [31,32]. Grafting arginines onto their surface was shown to enhance the plasmid DNA transfection efficiency of PAMAM or polypropyleneimine dendrimers [33,34]. Substituting terminal cationic groups of polylysine dendrimers with arginine, but not histidine, also increased plasmid transfection efficiency. Similarly, an arginine-terminated amphiphilic dendrimer exhibited improved siRNA delivery compared to the corresponding non arginine-decorated control dendrimer [35]. Overall, the literature is rich in studies showing enhanced dendrimer entry conferred by surface arginine residues, but indicates that this is not mediated via caveola entry. Nystatin treatment of cells does not impair entry of arginine-rich peptides [36], while methyl-*β* cyclodextrin treatment increases, rather than prevents, octa-arginine peptide entry [37]. Cellular uptake of these peptides was suggested to rely on cell surface sulphated polysaccharides, possibly via electrostatic interaction [36], followed by macropinocytosis with F-actin reorganization [38]. Moreover, caveolae do not mediate the endocytosis advantage conferred by arginine residues to amphiphilic dendrimers [35] since the increased siRNA delivery was demonstrated with PC3 prostate cancer cells, which do not express PTRF and do not form caveolae [18]. Further studies are warranted to unveil the mechanism by which arginine surface residues promote asymmetric peptide dendrimers endocytosis in cells that harbour caveolae, and to test whether this holds true when the dendrimers carry therapeutic cargo.

## Conclusions

The presence of caveolae stimulates entry of cationic dendrimers in cells and dendrimer structure (positive charge density and arginine head groups) can be modified to promote endocytosis in cells that form caveolae.

## Supporting Information

S1 Fig RP-HPLC elution profiles. For analytical RP-HPLC, a C_18_ column with mobile phase; Solvent A- distilled water with 0.1% v/v TFA and Solvent B- CH_3_CN_(aq)_ with 0.1% v/v TFA was used. The analysis of each purified dendrimer was performed at 1 mL/min flow rate with 100% Solvent B (0–20 min). A blank chromatogram reading zero was run between each sample.
(DOCX)

S2 Fig Evaluation of cell viability after exposure to 16 charged cationic arginine head group dendrimer. WT, caveolin-1 KO and PTRF cells were exposed to the indicated concentrations of dendrimer for 12 h. Viability was assessed using the MTT assay. Results are expressed as percentage cell viability compared to untreated cells and shown as mean ± S.E.M. (n = 3 separate experiments), ns; not significant, **p<0.01 (one way ANOVA).
(PPTX)

S3 Fig Visualisation of cellular uptake of cationic dendrimer. WT iMEFs were incubated with 16 charged cationic arginine head group dendrimer (10 μg/ml) for 12 h. Cells were fixed and biotinylated dendrimer was visualised using Cy3-streptavidin and nuclei sained with DAPI. A) Representative fluorescence microscopy image at 63X magnification. B) Representative confocal fluorescent xy section at 60X magnification. Bar in both images represents 20 μm.
(PPTX)

S4 Fig Representative images of cellular uptake of dendrimers. A) WT iMEFs were incubated with 8 charged cationic anionic or neutral head group dendrimer for 12 h. B) WT, Cav-1 KO or PTRF KO iMEFs were incubated with 16 charged cationic Arg head group dendrimer for 12 h. Cells were fixed and biotinylated dendrimer was visualised using Cy3-streptavidin and nuclei sained with DAPI. Representative fluorescence microscopy images at 20X magnification
(TIF)
