# Supplementary material for: Molecular Determinants of the Cellular Entry of Asymmetric Peptide Dendrimers and Role of Caveolae
Source: PLoS One. 2016 Jan 20;11(1):e0147491. doi: 10.1371/journal.pone.0147491 (PMC4720277; doi:10.1371/journal.pone.0147491)

**Supplementary Figure 1**

**4+Arg**


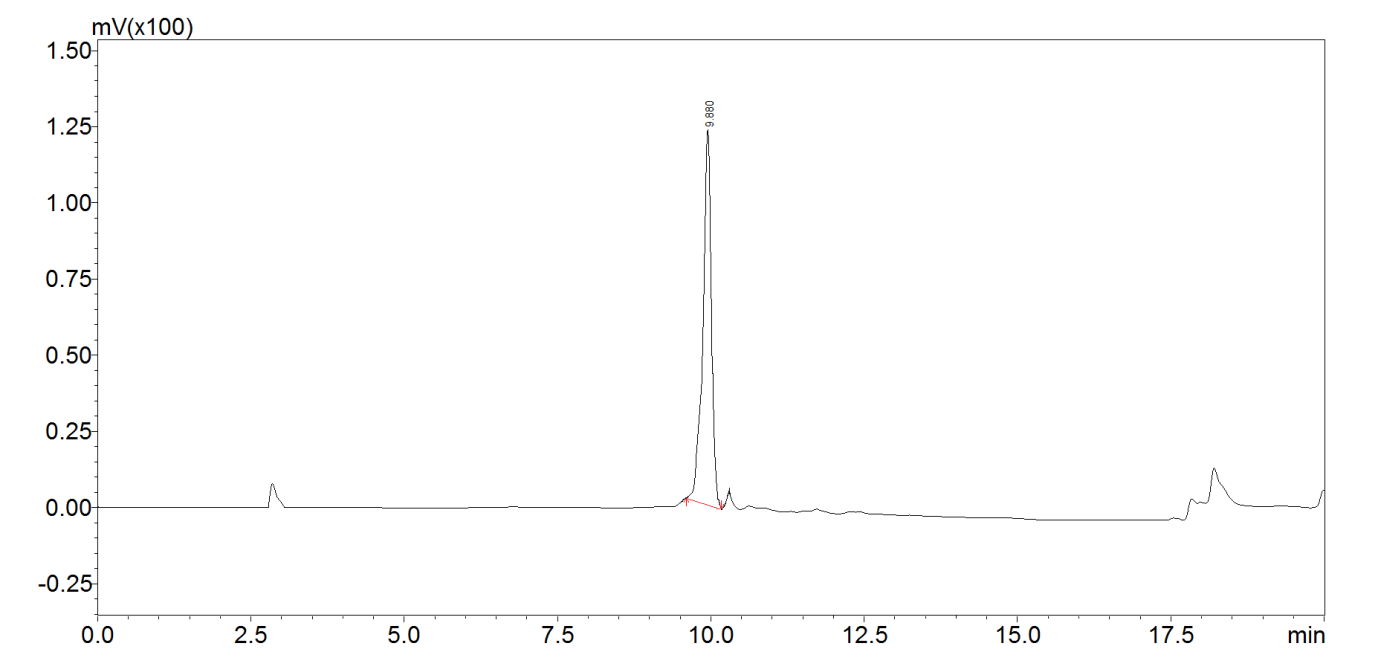


**4+Lys**


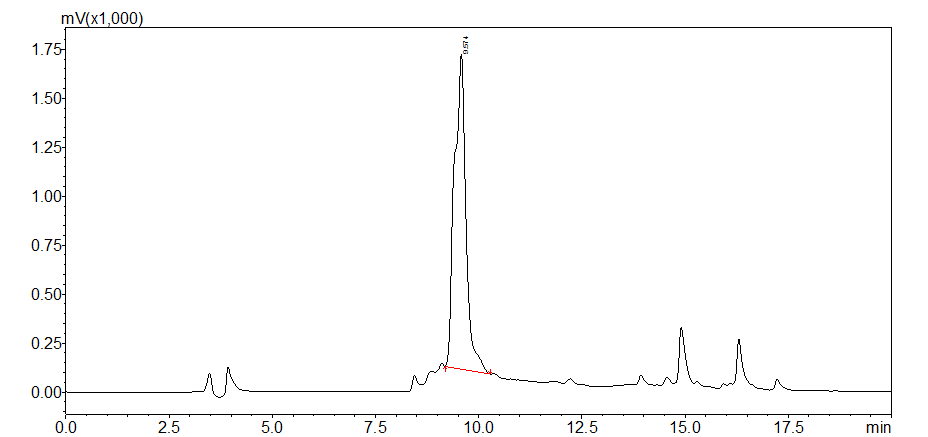


**4+His**


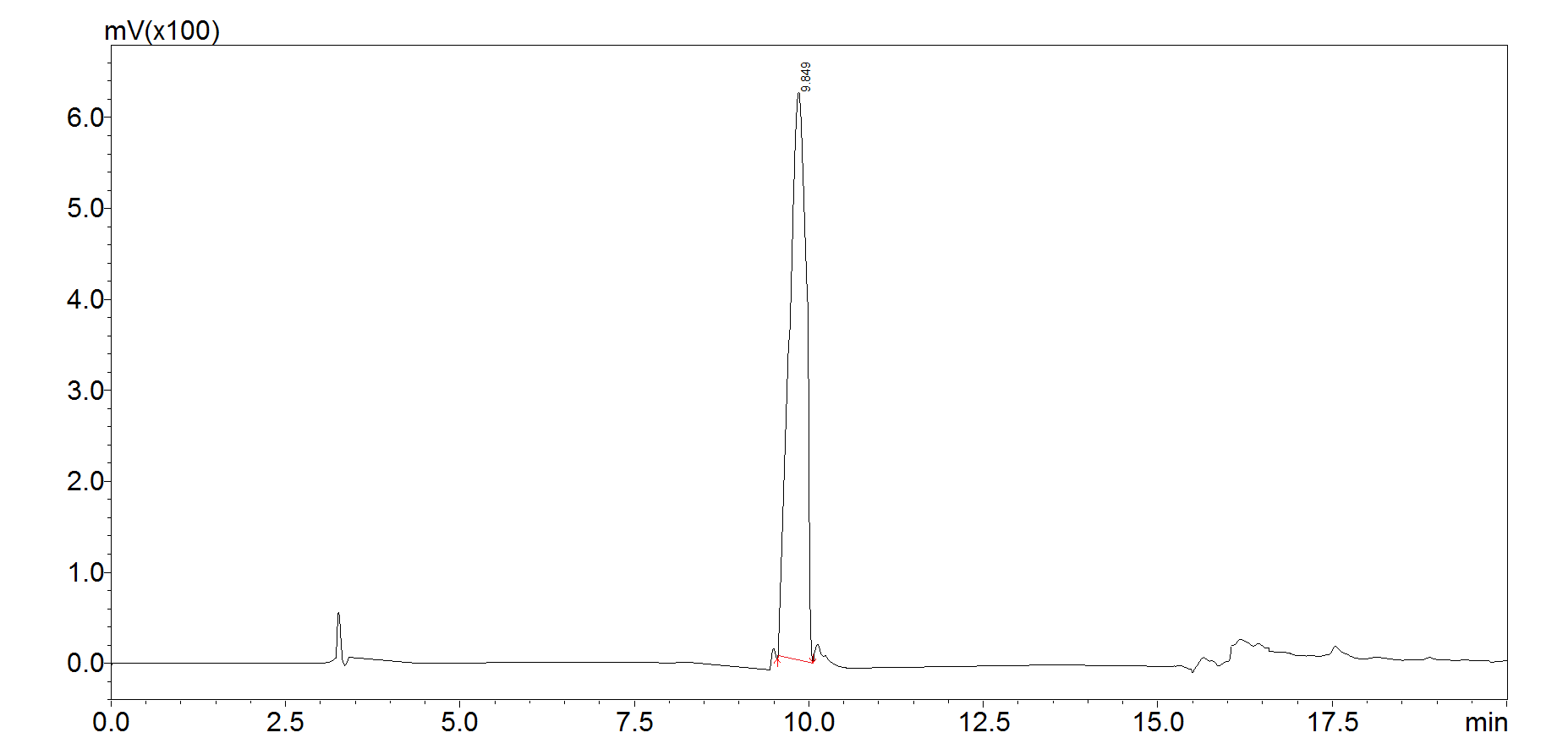


**8+Arg**


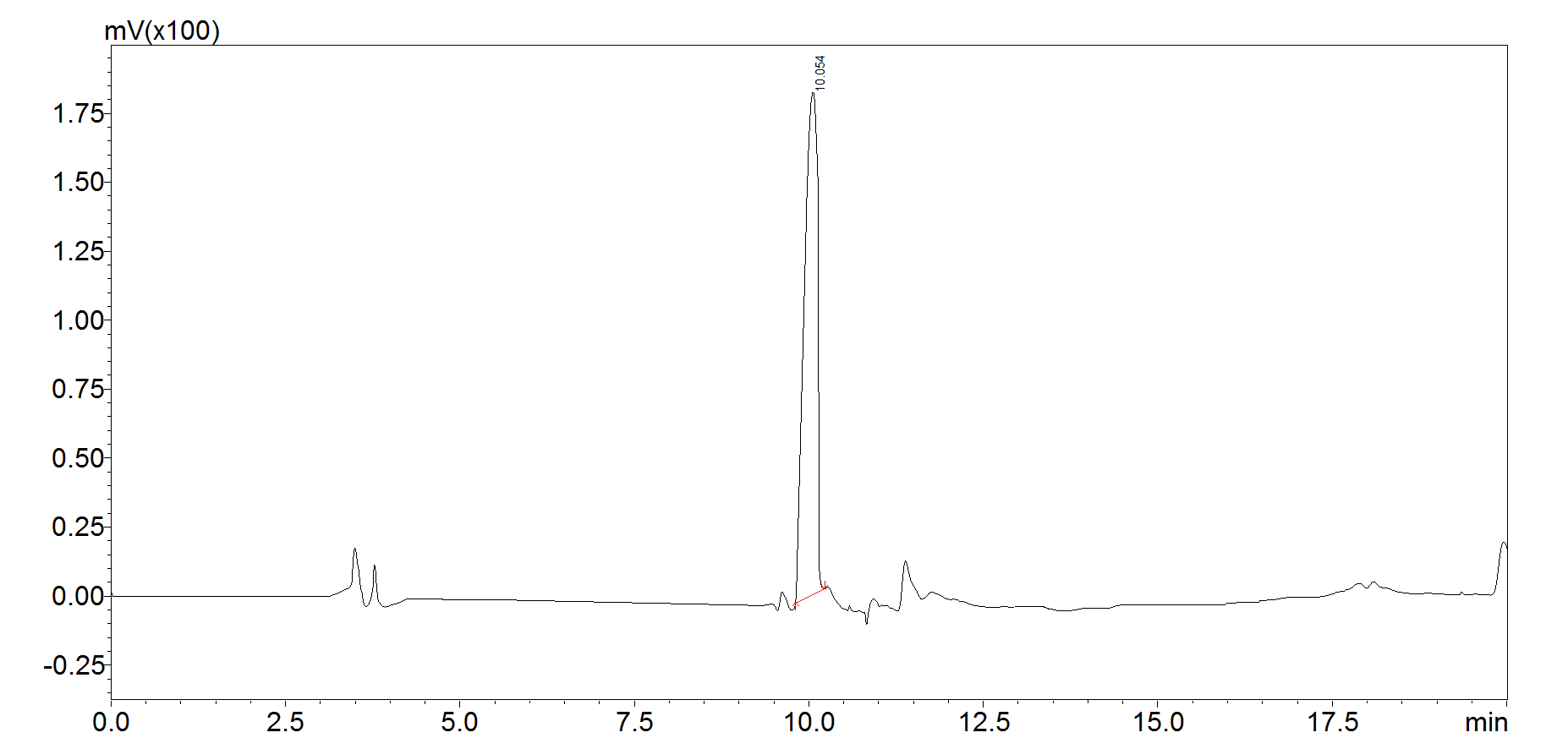


**8+Lys**


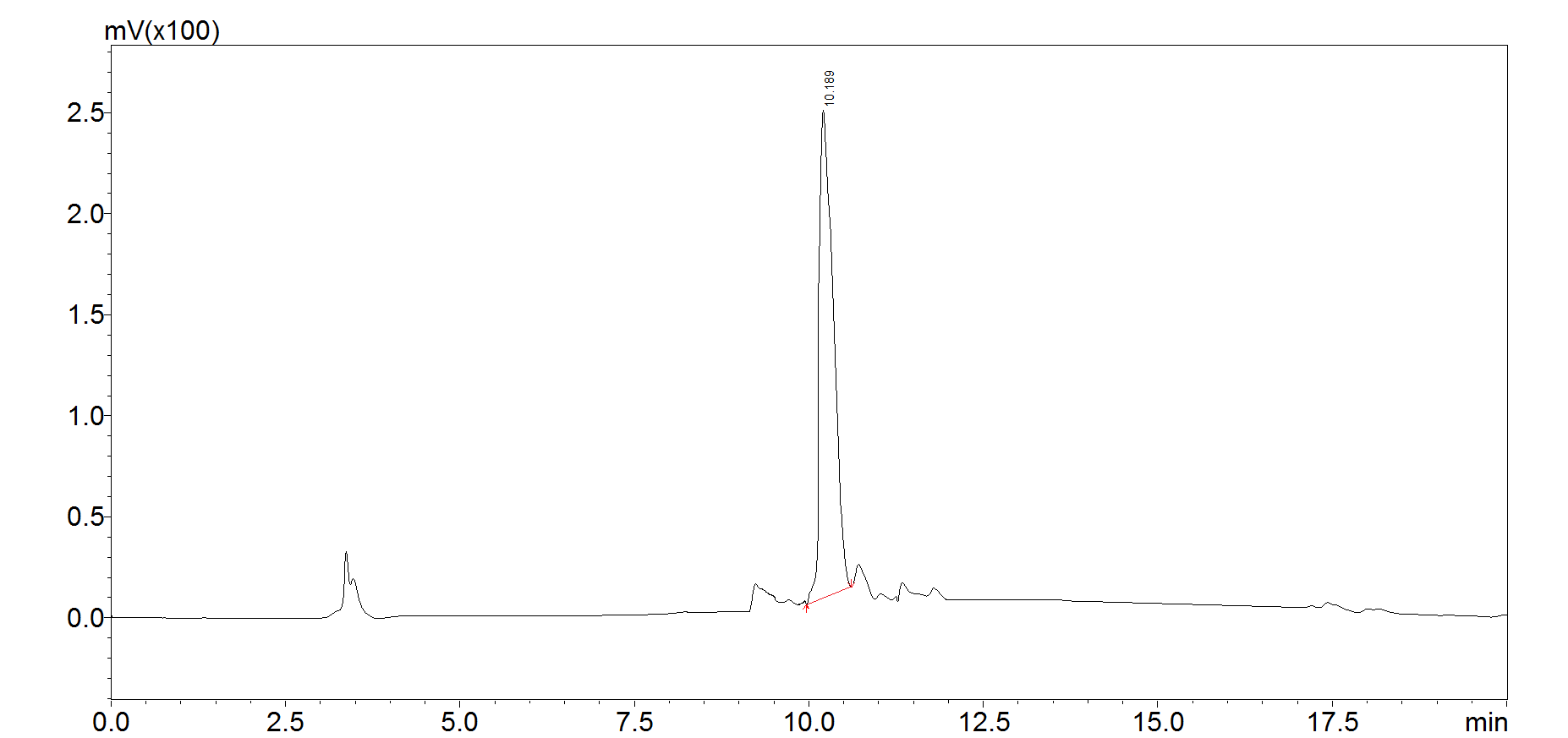


**8+His**


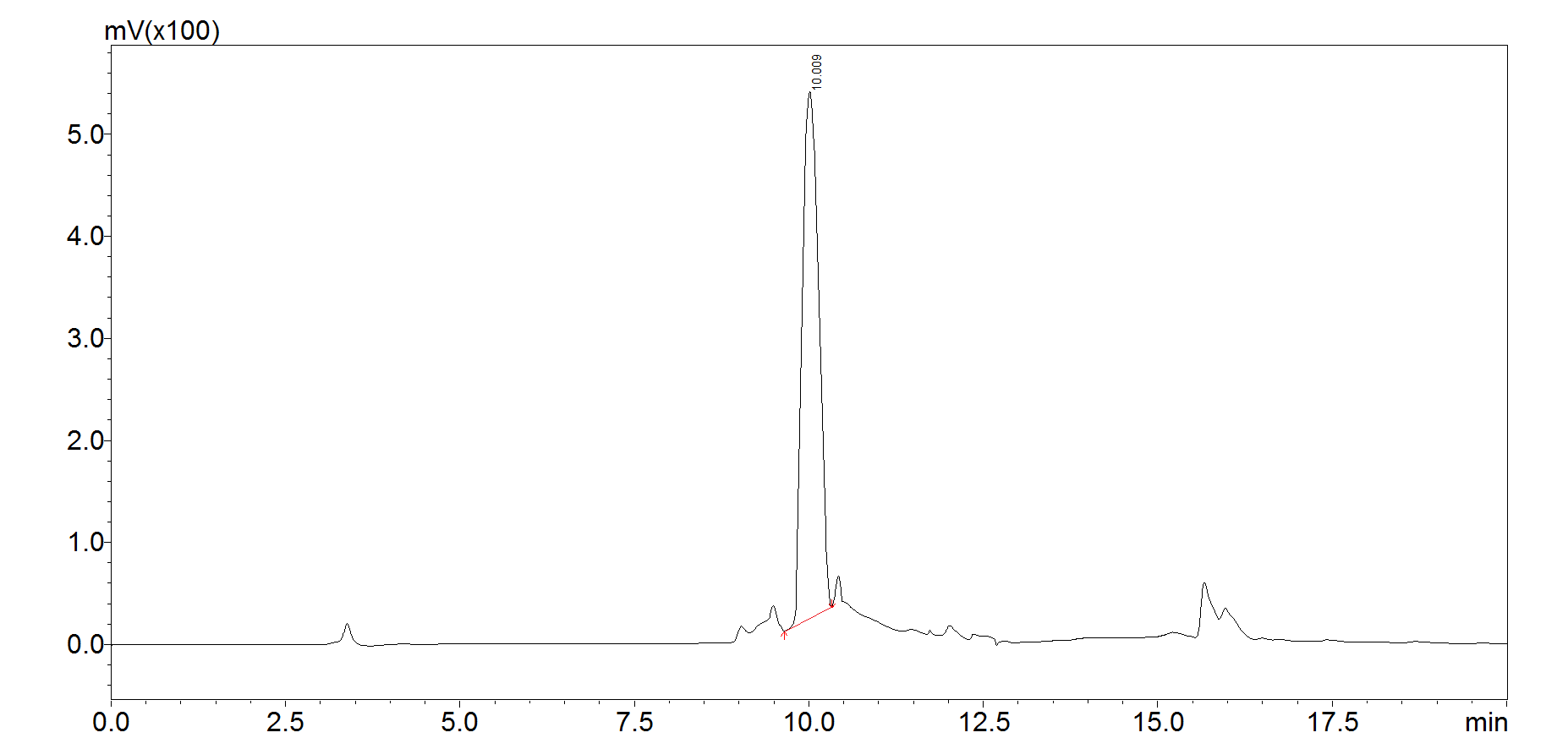


**16+Arg**


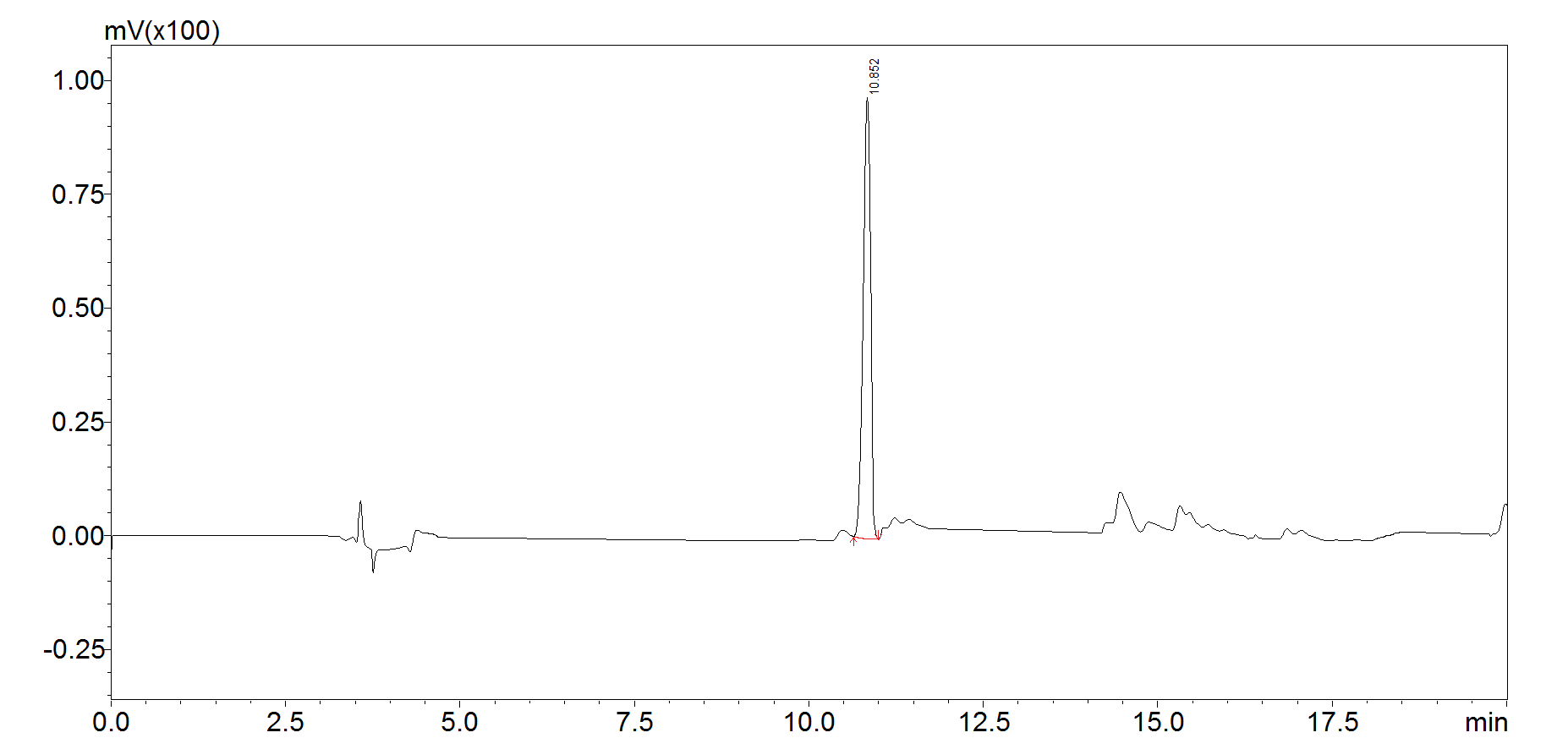


**16+Lys8**


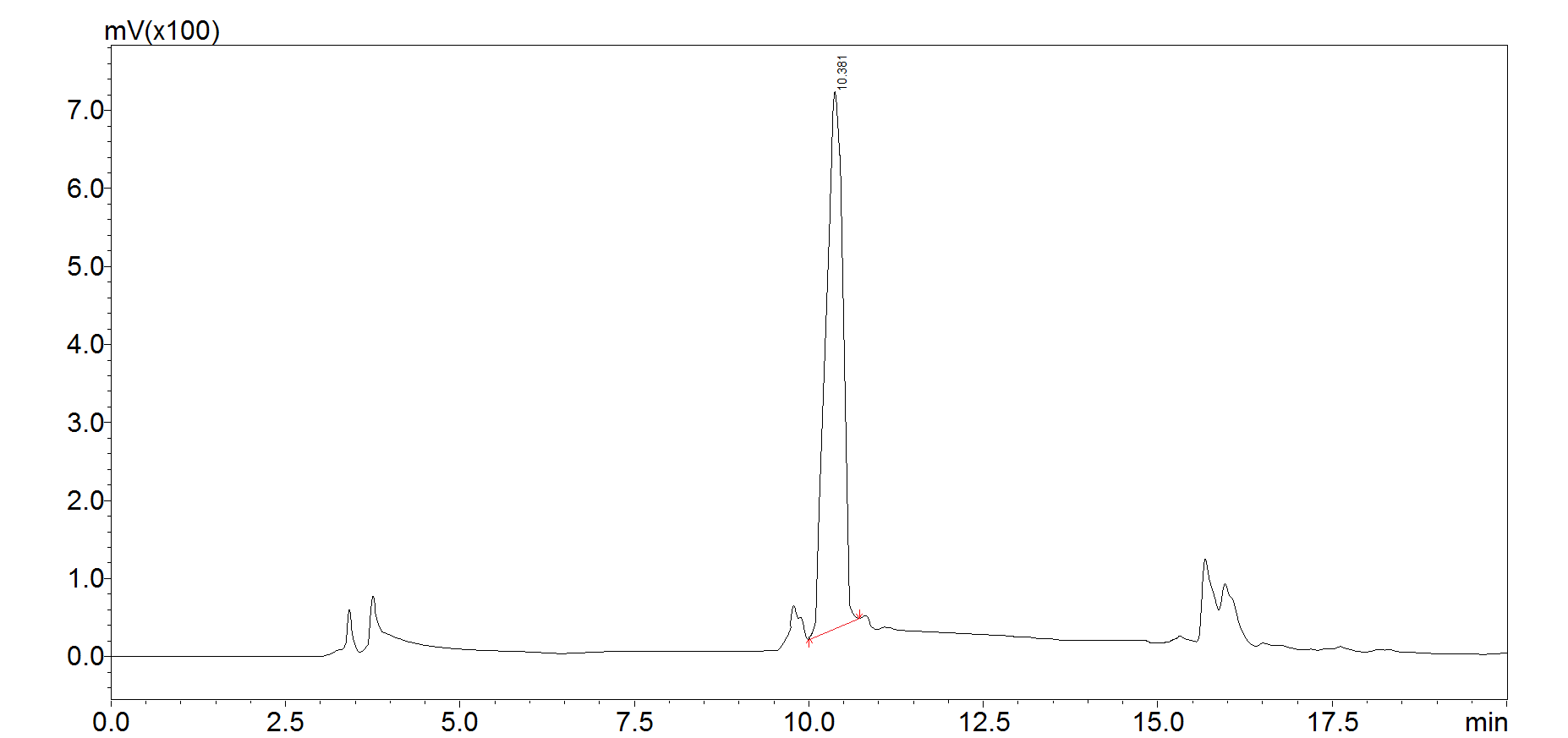


**16+His**


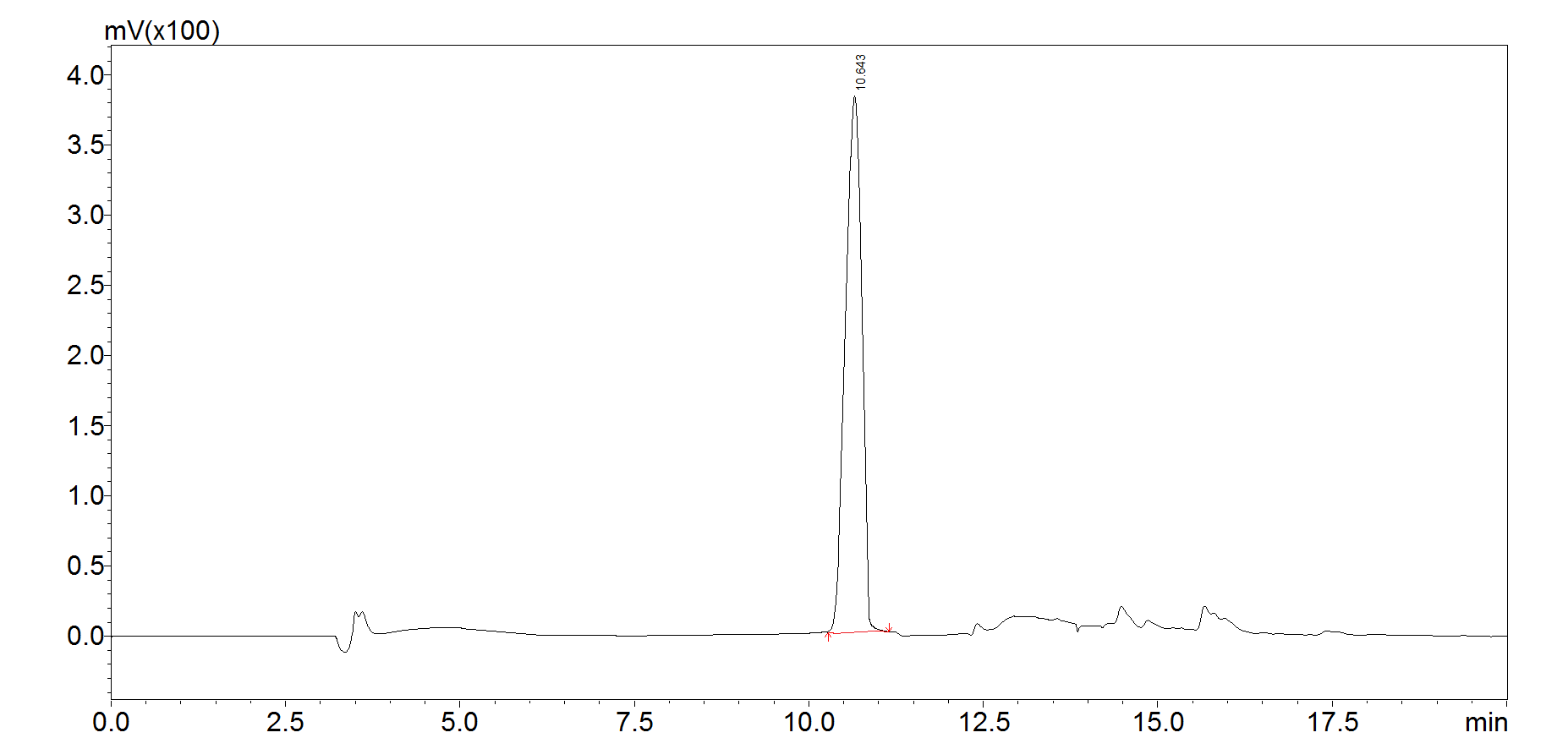


**Anionic dendrimer**


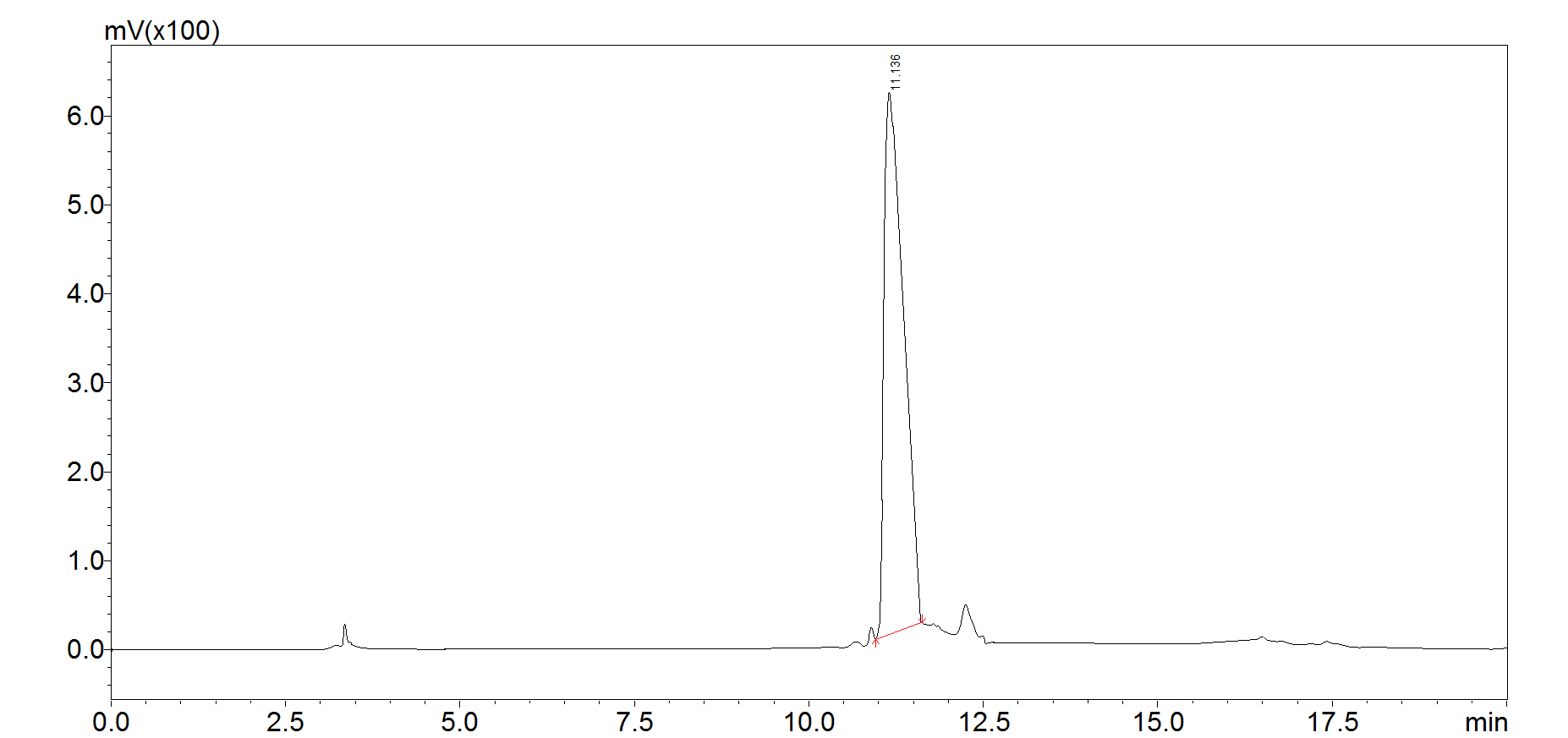


**Neutral capped dendrimer**


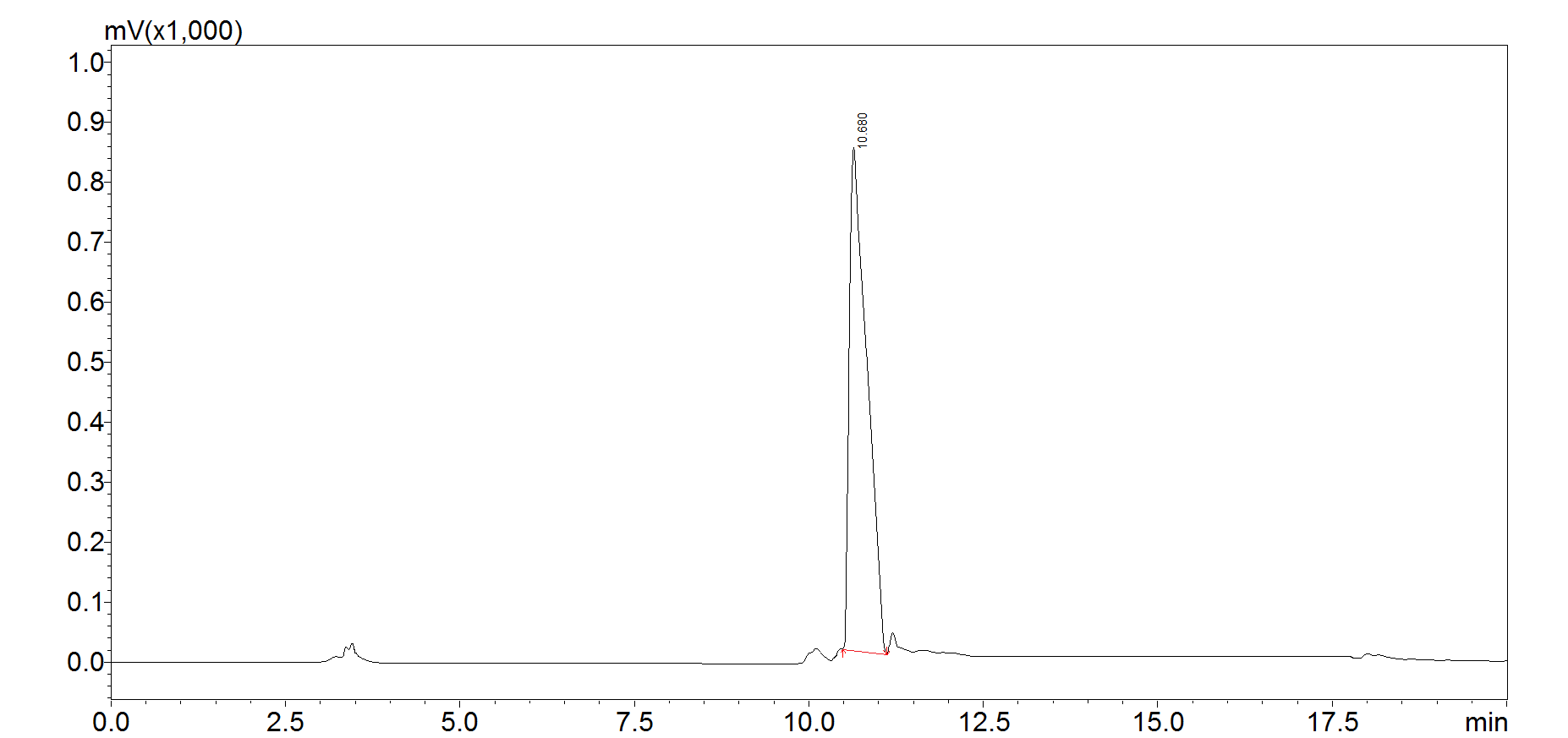

Supplement: S1 Fig — For analytical RP-HPLC, a C18 column with mobile phase; Solvent A- distilled water with 0.1% v/v TFA and Solvent B- CH3CN(aq) with 0.1% v/v TFA was used. The analysis of each purified dendrimer was performed at 1 mL/min flow rate with 100% Solvent B (0–20 min). A blank chromatogram reading zero was run between each sample. (DOCX) [file pone.0147491.s001.docx]
