# Supplementary figures and images for: Molecular Determinants of the Cellular Entry of Asymmetric Peptide Dendrimers and Role of Caveolae
Source: PLoS One. 2016 Jan 20;11(1):e0147491. doi: 10.1371/journal.pone.0147491 (PMC4720277; doi:10.1371/journal.pone.0147491)

## Slide 1
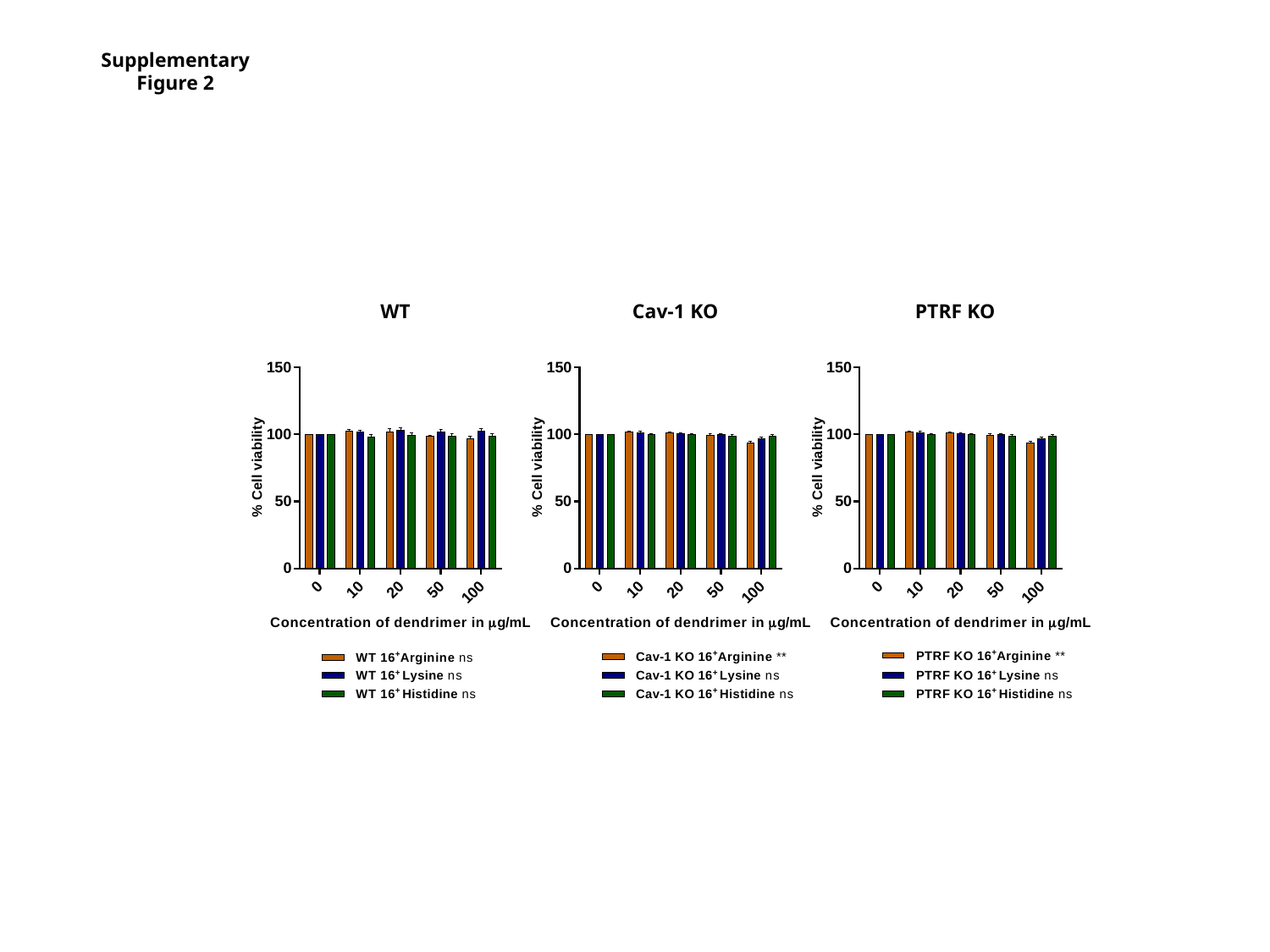

Supplementary Figure 2
WT
Cav-1 KO
PTRF KO

Supplement: S2 Fig — WT, caveolin-1 KO and PTRF cells were exposed to the indicated concentrations of dendrimer for 12 h. Viability was assessed using the MTT assay. Results are expressed as percentage cell viability compared to untreated cells and shown as mean ± S.E.M. (n = 3 separate experiments), ns; not significant, **p<0.01 (one way ANOVA). (PPTX) [file pone.0147491.s002.pptx]

## Slide 1
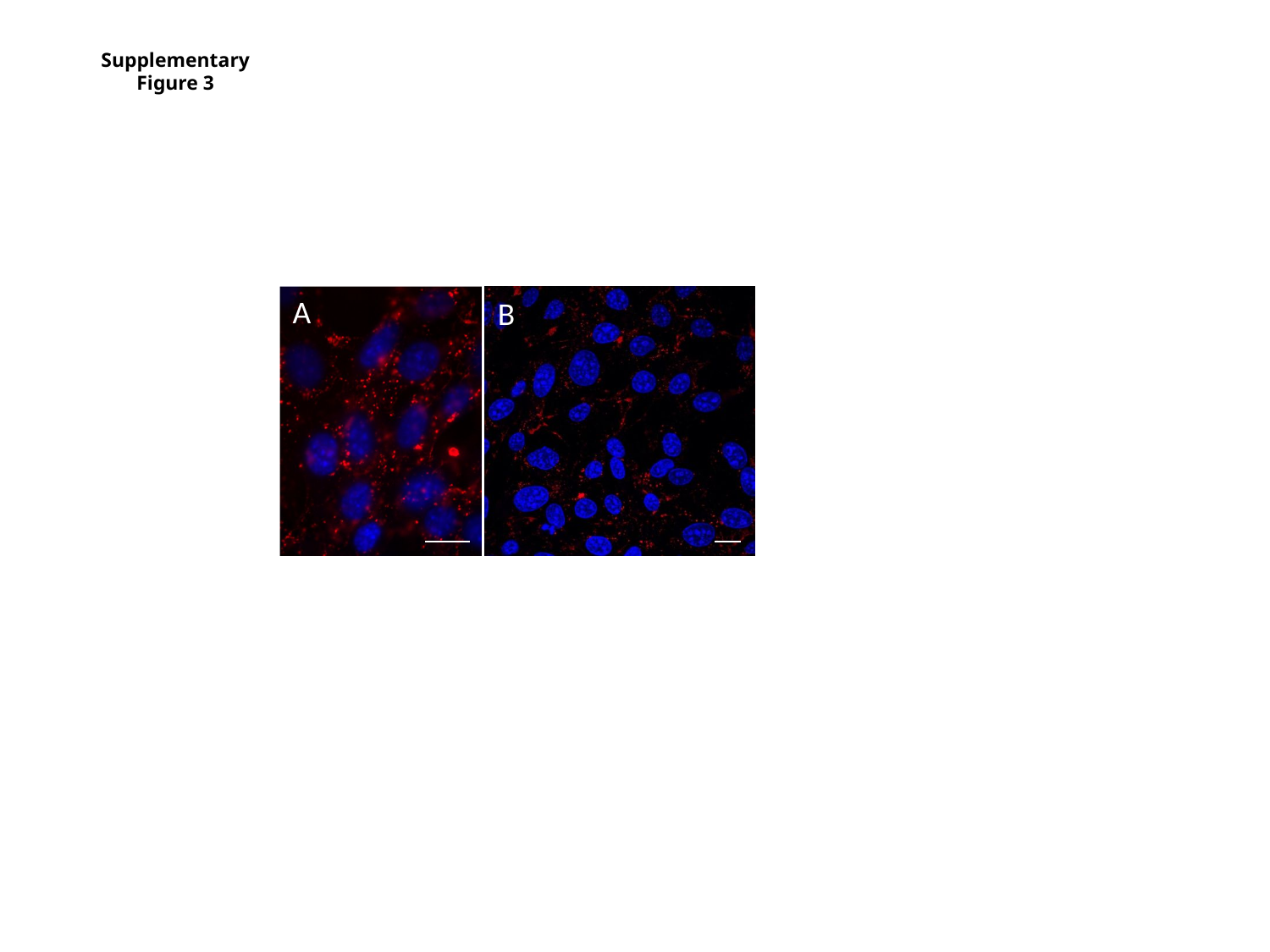

Supplementary Figure 3
B
A

Supplement: S3 Fig — WT iMEFs were incubated with 16 charged cationic arginine head group dendrimer (10 μg/ml) for 12 h. Cells were fixed and biotinylated dendrimer was visualised using Cy3-streptavidin and nuclei sained with DAPI. A) Representative fluorescence microscopy image at 63X magnification. B) Representative confocal fluorescent xy section at 60X magnification. Bar in both images represents 20 μm. (PPTX) [file pone.0147491.s003.pptx]

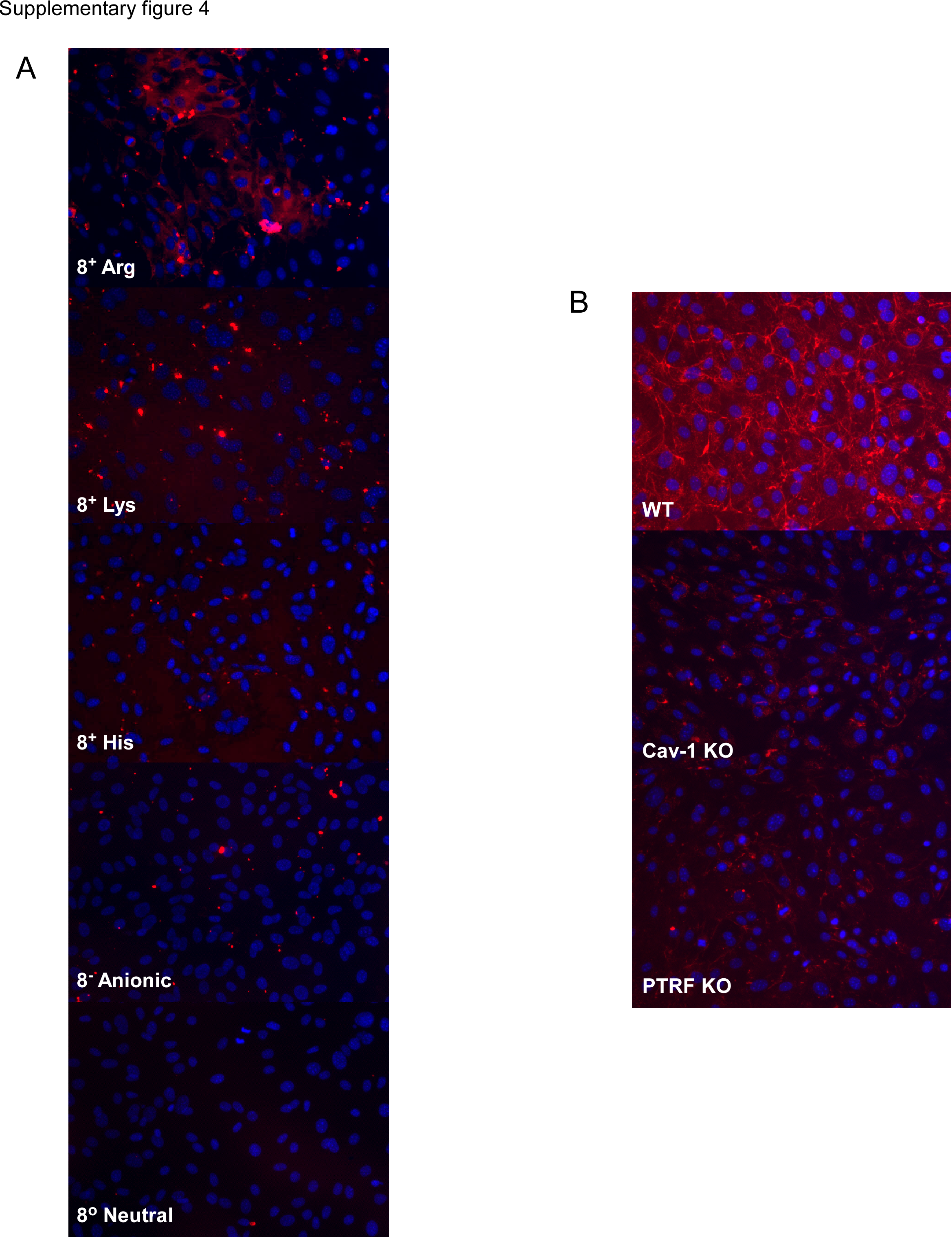

Supplement: S4 Fig — A) WT iMEFs were incubated with 8 charged cationic anionic or neutral head group dendrimer for 12 h. B) WT, Cav-1 KO or PTRF KO iMEFs were incubated with 16 charged cationic Arg head group dendrimer for 12 h. Cells were fixed and biotinylated dendrimer was visualised using Cy3-streptavidin and nuclei sained with DAPI. Representative fluorescence microscopy images at 20X magnification (TIF) [file pone.0147491.s004.tif]
